# Nurses' Contributions.—First Instalment

**Published:** 1920-02-21

**Authors:** 


### "DAILY TELEGRAPH" SHILLING FUND.

### NURSES' CONTRIBUTIONS.—First Instalment.

**The following contributions to the Shilling Fund for Nurses are announced in the "Daily Telegraph" of February 18 :—**

Shillings.

Nightingale Training School, St. Thomas's Hospital 5,317

Royal Free Hospital ... ... ... ... ... ... 1,600

Members of Nursing Staff, 1st London General Hospital, T.F., Camberwell ... ... ... ... ... 1,000

Members of College of Nursing, London Centre, 1,173s. 6d.; Nursing Staff, Hackney Infirmary, 30s.; Kensington Infirmary, 25s. 6d.; Paddington, 41s. ... 1,270

Norfolk and Norwich Centre of the College of Nursing, Ltd.:
Norfolk and Norwich Hospital, Norwich, 500s.; from the Norfolk and Norwich Eye Infirmary, 67s.; ditto from the Jenny Lind Hospital for Children, Norwich, 33s.; from the Infirmary, Norwich, 32s.; from the Yarmouth Hospital of Great Yarmouth, 20s.; from the Bethel Hospital, Norwich, 10s.; from the Cavell Home, Norwich, 8s.; from Other Nurses of the Norfolk and Norwich Centre, College of Nursing, Ltd., 7s.; Mrs. Branford Morgan, Norwich, 42s.; Mrs. Harker, 20s.; Mrs. G. C. Chittock, Norwich, 42s. ... ... ... ... ... 781

Matron and Nursing Staff, Lake Hospital, Ashton-under-Lyne ... ... ... ... ... ... 700

Liverpool Centre, College of Nursing ... ... ... 657

Edinburgh Centre of the College of Nursing ... 640 6

Plymouth Hospital and Institution and Members of the College of Nursing, Plymouth Centre ... ... 402

Nursing Staff of the Leicester Royal Infirmary ... 400

East Lancs College of Nursing, Manchester:
From the Nurses of the Royal Infirmary, the Royal Eye Hospital, Barnes Convalescent Hospital, Hope Hospital, Ladywell Sanatorium, General Hospital, Stockport; General Hospital, Ashton-under-Lyne; Royal Infirmary, Oldham; Stepping Hill Infirmary; the District Nursing Homes of Salford, Bradford, Manchester, Harpurly, Plymouth Grove, and Enfield Nursing Home ... ... ... ... ... ... 409

Collected by the Yorkshire Centre of the College of Nursing ... ... ... ... ... ... 408

The Lincoln County Hospital, Staff, and Friends 260
Per Lincoln City Hospital ... ... ... ... 43
Per Grimsby Hospital ... ... ... ... ... 10—313

Royal Hampshire County Hospital, Winchester ... 290 6

Per Brighton and Hove Centre, College of Nursing:
Royal Sussex County Hospital ... ... ... 100
New Sussex Hospital ... ... ... ... ... 12
Sister Pierson and Friends ... ... ... ... 11
Nurse Strachan ... ... ... ... ... ... 70
Nurse Owens ... ... ... ... ... ... 20
Royal Alexander Hospital ... ... ... 10—223

Nurses and their Friends at Addenbrooke's Hospital, Aberdeen ... ... ... ... ... ... 251

Staff of the Hospital for Consumption, Brompton ... 202

Amount collected from and by the members of the Aberdeen Centre of the College of Nursing ... ... 200

Some Nurses of Hull ... ... ... ... 132 6

Queen Victoria's Jubilee Institute—The Superintendents and Staff of the Queen's Nurses' Homes:
Brighton ... ... ... ... ... ... 10
Bury ... ... ... ... ... ... 6
Central St. Pancras ... ... ... ... 4
Exeter ... ... ... ... ... ... 7
Halifax ... ... ... ... ... ... 5
Portsmouth ... ... ... ... ... 8
Southampton ... ... ... ... ... 5
Kilburn ... ... ... ... ... ... 6
51
Queen's Nurses ... ... ... ... ... 73 9
124 9

Given and Collected by the Nursing Staff of the Royal Cornwall Infirmary, Truro ... ... ... ... 122

Nursing Staff, General Hospital, Birmingham ... 123

Matron and Nursing Staff of the Crumpsall Infirmary, Manchester ... ... ... ... ... ... 120

Nursing Staff of the Middlesex Hospital ... ... 120

Nursing Staff of the North Evington Infirmary, Leicester ... ... ... ... ... ... 110

From the Nursing Staff of the Dreadnought Hospital ... ... ... ... ... ... 60
Nursing Staff of the Royal Herbert Hospital ... 50
110

Dame Sidney Browne, Matron-in-Chief, T.F.N.S. ... 100

Matron and Nursing Staff of the Great Northern Central Hospital ... ... ... ... ... 100

Bromhead Nursing Institute, Lincoln Centre ... ... 100

Shillings.

Matron and Nursing Staff, North Staffordshire Infirmary, Stoke-on-Trent ... ... ... ... ... 100

Miss F. Trevor Gray, in memory of Sister C., drowned on duty in Mesopotamia ... ... ... ... 100

Dame Sarah Swift, Matron-in-Chief, British Red Cross 100

Nurses at Archer House, Ramsgate ... ... ... 100

3rd London General Hospital, Wandsworth and Chelsea Infirmary ... ... ... ... ... ... ... 109

**Sums under 100s.:**
Collected by Nurse Crosbie, permanently injured in air raid, 71s. 6d.; Kent and Canterbury Hospital, Canterbury, 60s.; Staff at St. Mary's, Paddington, 89s.; Salisbury Infirmary, 60s.; Matron, 20s.; Nursing Staff of the Salford Royal Hospital, 72s.; Nurse Galledge, Bristol, 70s.; Nursing Staff of the Coventry and Warwickshire Hospital, 66s.; a Few of the Nursing Staff at Chesterfield Place, Bristol, 67s.; some Members of the Nursing Staff at the North-Eastern Hospital, South Tottenham, 62s.; the Nursing Staff of the Royal Halifax Infirmary, Halifax, 56s.; Nurses of the London Homœopathic Hospital, 51s. 6d.; Matron and Nurses of Hampstead General and North-West London Hospital, Haverstock Hill, N.W., 50s.; Nurses of Shirley Warren Infirmary, Southampton, 50s.; Nursing Staff, 25 Sussex Square, Brighton, 46s.; Whitechapel Infirmary Nursing Staff, 40s.; Nursing Staff of Bethnal Green Infirmary, 30s.; Donation from Ancoats Hospital, East Lancs, 25s.; Nursing Staff of the Royal South Hants and Southampton Hospital, 33s.; the Nurses at the Royal Salop Infirmary, Shrewsbury, 26s.; Greenwich Union Infirmary, 22s.; Nursing Staff, South London Hospital for Women, 80s.; Staff of Nursing Home, Stoke-on-Trent, 40s. 6d.; Northampton General Hospital, 30s. ... ... ... ... ... ... ... 1,217 6

**Sums of 21s. and under:**
C. A. Tait McKay, late Matron 4th S.G. Hospital, Plymouth, 21s.; Nursing Staff of the Infirmary, London Road, Coventry, 21s.; "A Silver Badge Nurse," 20s.; Nurse A. H. Denny, member of the College of Nursing, 20s.; the General Infirmary, Macclesfield, 20s.; from a Grateful Patient at Kettering General Hospital, 20s.; Nurse Mary Breeze, Birmingham, 20s.; Helen Scott (from a patient), 20s.; Nurse Ducker, Limerick, 13s.; Nurse Eldridge, Tring, 12s. ... ... ... ... ... 187 0

**Sums of 10s. and under:**
Three Nurses, 10s.; Nurse Thompson, Bristol, 10s.; Nurse Furnival, 10s.; Matron and Members of Nursing Staff of Central London Ophthalmic Hospital, St. Pancras, 10s.; Superintendent and Six Nurses of the Infirmary of the North Brierley Union, Clayton, and Members of Yorkshire Branch of College of Nursing, 10s.; Matron and Sister, Sussex Throat and Ear Hospital, Brighton, 10s.; Nurse F. Smith, Hackney, 10s.; Hartley Jam Factory, per Nurse Croslie, 8s.; Nurse Taylor, Stoke-on-Trent, 7s.; Nurses of Eltham and Mottingham Cottage Hospital, 7s. ... ... ... ... ... ... ... ... 92 0

**Sums of 5s. and under:**
Mabel Dent, Nurse, 5s.; B. C. F. C. C., 5s.; Nurse A. E. Butler, Alderbury, 5s.; F. Addy, the Infirmary, Alcester, 5s.; the Matron of the Hove Hospital, 5s.; the Matron of the Newe Hospital, Edgbaston, 5s.; the Matron of the Hospital, Stratford-on-Avon, 5s.; Miss Holloway, Victoria Nursing Institution, Walsall, 5s.; Olive M. Bros, Gravelly Hill, 3s.; Sister B. F. Bourne, St. Albans, 3s.; M. T., 3s.; Nurse Herbert, 3s.; Nurse A. Challoner, 2s. 6d.; Sister T., Norwich, 2s. 6d.; Nurse Moi, 2s. 6d.; Nurse Richford, 2s. 6d.; Sister Dale, U.C.H., 2s.; A Nurse, Eastbourne, 2s.; F. M. L., Nurse for Thirty Years, 2s.; College Member, Norah, 2s.; Nurse Hardy, Bournemouth, 2s.; J. and K. Tyrrell, Eastbourne, 2s.; Nurse Roe and Arnold, Longwood, 2s.; A. M. T., 2s.; "From a Nurse, 2s.; Sister H., 2s. 6d. ... ... ... 82 6

**Sums of 1s.:**
A Nurse; Sister B.; Nurse M. Clarke; A Nurse; Nurse Frances A. Dunn; Nurse M. Entwisle, Bournemouth; E. B., Leeds Centre College of Nursing; Nurse Ingram, Ballater (N.B.); Nurse F. C. M. Lindsay; Miss M. J. L. Lyons; Nurse Meredith, Manchester; Nurse Stokes, U.C.H.; Sister Barry Sullivan, Hinckley; Nurse Scott, Nurse M. Parkinson, Lytham; Miss K. N. Wooler, late Sister, T.F.N.S. ... ... ... ... ... 16 0

**Total, 18,421 shillings.**

